# Genome-wide analysis of *NPR1*-like genes in citrus species and expression analysis in response to citrus canker (*Xanthomonas axonopodis* pv. *citri*)

**DOI:** 10.3389/fpls.2024.1333286

**Published:** 2024-03-25

**Authors:** Mobeen Ali, Muhammad Shafiq, Muhammad Zeshan Haider, Adnan Sami, Pravej Alam, Thamir Albalawi, Zuha Kamran, Saleh Sadiq, Mujahid Hussain, Muhammad Adnan Shahid, Mouna Jeridi, Ghulam Abbas Ashraf, Muhammad Aamir Manzoor, Irfan Ali Sabir

**Affiliations:** ^1^ Department of Horticulture, Faculty of Agricultural Sciences, University of the Punjab, Lahore, Pakistan; ^2^ Department of Plant Breeding & Genetics, Faculty of Agriculture Sciences, University of the Punjab, Lahore, Pakistan; ^3^ Department of Biology, College of Science and Humanities in Al-Kharj, Prince Sattam Bin Abdulaziz University, Al-Kharj, Saudi Arabia; ^4^ Horticultural Science Department University of Florida-Institute of Food and Agricultural Sciences (IFAS) North Florida Research and Education Center, Gainesville FL, United States; ^5^ Biology Department, College of Science, King Khalid University, Abha, Saudi Arabia; ^6^ College of Environment, Hohai University, Nanjing, China; ^7^ School of Agriculture and Biology, Shanghai Jiao Tong University, Shanghai, China; ^8^ College of Horticulture, South China Agricultural University, Guangzhou, China

**Keywords:** citrus, expression profile of *NPR1*, phylogenetic analysis, salicylic acid, biotic stress

## Abstract

Citrus fruits, revered for their nutritional value, face significant threats from diseases like citrus canker, particularly impacting global citrus cultivation, notably in Pakistan. This study delves into the critical role of *NPR1*-like genes, the true receptors for salicylic acid (SA), in the defense mechanisms of citrus against *Xanthomonas axonopodis* pv. *citri* (Xcc). By conducting a comprehensive genome-wide analysis and phylogenetic study, the evolutionary dynamics of *Citrus limon* genes across diverse citrus cultivars are elucidated. Structural predictions unveil conserved domains, such as the BTB domain and ankyrin repeat domains, crucial for the defense mechanism. Motif analysis reveals essential conserved patterns, while cis-regulatory elements indicate their involvement in transcription, growth, response to phytohormones, and stress. The predominantly cytoplasmic and nuclear localization of *NPR1*-like genes underscores their pivotal role in conferring resistance to various citrus species. Analysis of the Ks/Ka ratio indicates a purifying selection of *NPR1*-like genes, emphasizing their importance in different species. Synteny and chromosomal mapping provide insights into duplication events and orthologous links among citrus species. Notably, Xac infection stimulates the expression of *NPR1*-like genes, revealing their responsiveness to pathogenic challenges. Interestingly, qRT-PCR profiling post-Xac infection reveals cultivar-specific alterations in expression within susceptible and resistant citrus varieties. Beyond genetic factors, physiological parameters like peroxidase, total soluble protein, and secondary metabolites respond to SA-dependent PR genes, influencing plant characteristics. Examining the impact of defense genes (NPR1) and plant characteristics on disease resistance in citrus, this study marks the inaugural investigation into the correlation between NPR1-associated genes and various plant traits in both susceptible and resistant citrus varieties to citrus bacterial canker.

## 1 Introduction

Globally, citrus fruits stand out as the predominant category of commercially cultivated fruit within the Rutaceae family, offering a wealth of nutritional benefits and playing a crucial role in the world economy (Russo et al., 2021). Unfortunately, the citrus plant faces a significant threat from various diseases such as greening, citrus decline, and gummosis (Malik et al., 2021). Among these is Citrus Canker Disease (CCKD), caused by *Xanthomonas axonopodis* pv. *citri* (Xcc), which poses a major risk to citrus crop yield and quality worldwide, with particular severity in Pakistan (Malamud et al., 2011; Sami et al., 2023a; Sami et al., 2023b). The Sargodha region’s citrus orchards contribute over 90% to Pakistan’s national citrus production. Yield losses in Pakistan due to citrus canker can range from 10% to 50%, depending on infection severity and environmental conditions (Sabir et al., 2010; Almas et al., 2023). However, their potential is hampered by the onslaught of canker, evidenced by symptoms like discoloration, abrasions, and water-soaked lesions on fruit, stems, or leaves (Shahbaz et al., 2023). Several commercially important citrus varieties, including grapefruit, sweet oranges [*C. sinensis C. sinensis* (L.) Osbeck], lemons [*C.limon* (L.) Burm. F.], and key lime (*Citrus aurantifolia* Swingle), are susceptible to bacterial canker (Graham et al., 2004) impacting both the quantity and quality of fresh and processed fruit. This, in turn, leads to substantial economic losses for citrus growers (Spreen et al., 2003). The economic repercussions extend beyond the orchards, as limited fruit trade among states or internationally from canker-affected regions takes a severe toll on the overall economy (Duan et al., 2009; Khan et al., 2023).

Several alternative strategies for disease management have been devised with the aim of establishing enduring solutions for disease control in the long term. These methods encompass plant defense mechanisms specifically crafted to counteract various stressors, encompassing attacks from pathogens (Kushalappa et al., 2016; Manzoor et al., 2023). Resistance genes are considered one of the major factors in defense mechanisms against pathogenic attacks. Resistance (R) genes often trigger downstream signaling responses during plant disease resistance (Malamud et al., 2011; Khan et al., 2023). An immune response, SAR (Shahbaz et al., 2022), promotes the exploration of the resistant gene family to an infectious pathogen. SAR is considered a prime factor for activating numerous pathogenesis-related (PR) genes, such as *NPR1* gene with its paralogues (*NPR3* and *NPR4*) (Fu and Dong, 2013). Exogenous application of some chemicals, like benzol 1,2,3-thiadiazole-7-carbothermic acid, 2,6-dichloroisonicotinic acid, salicylic acid, and S-methyl ester on plants are considered to be responsible for the stimulation of the SAR system (Durango et al., 2013). Additionally, it is noted that during specific hypersensitive responses, the signal transduction pathway(s) connecting HR to SAR involves the endogenous production of SA (Cao et al., 1997).

Based on structural motifs, transmembrane areas, interleukin-1 receptor domain, leucine rich repeat (LRR) domain, coiled coil (Spoel et al., 2003) domains, and nucleotide-binding sites (NBSs), resistant proteins are grouped into numerous superfamilies. The two most prevalent R genes (NBS-LRR) found in plants (Meyers et al., 2003) are further divided into sub-groups based on N-terminal CC or TIR domain (Li et al., 2018). *NPR1* genes and their homologues were revealed to regulate the resistance of disease in the citrus family against various pathogens (Peng et al., 2021). NONEXPRESSOR OF PATHOGENESIS-RELATED GENES 1 (*NPR1*, along with their NPR3 and NPR4 as paralogues) serves as authentic SA receptors, actively participating in both local and systemic immunity by regulating SA-mediated processes (Birkenbihl et al., 2017; Sami et al., 2023c).

The investigation of the resistant gene family across various citrus species is vital in light of the aforementioned findings (Ahmad et al., 2023; Hussain et al., 2023). Thus, this resistant gene family was recognized and analyzed thoroughly in different species of citrus (*Citrus sinensis*, *Citrus reticulata*, *Citrus fortunella*, *Citrus maxima*, *Citrus medica*, *Citrus ichangenesis*, *Atlanta buxifolia*, and *Poncirus trifoliata*) that ranged from susceptible to highly resistant against canker. Phylogenic classification, characterization on molecular basis conserved motifs, residues of amino acids, distribution on chromosomes, and composition of protein domains were investigated. Furthermore, the stress-related expression pattern of citrus-resistant genes in different regions was also analyzed using citrus species sequencing of RNA (RNA-seq) datasets (Haider et al., 2023b). The findings underscore the need for substantial support to facilitate applied research and in-depth exploration of resistant genes in citrus species and related plant families. This exploration holds promise for influencing the creation of disease-resistant citrus varieties using various breeding methods such as double haploid technology, hybridization, tissue culture, and backcrossing.

## 2 Materials and methods

### 2.1 Sequence data sources retrieval

Data pertaining to the amino acid sequences of *C. sinensis*, *C. reticulata*, *C. fortunella*, *C. limon*, *C. maxima*, *C. ichangenesis*, *C. maxima*, *P. trifoliata*, *C. climentina*, *A. buxifolia*, and *C. medica* were obtained from Citrus Genome database (https://www.citrusgenomedb.org). To confirm the presence of *NPR1*-like domains, retrieved amino acid sequences were subjected to searches at the SMART (http://smart.embl-heidelberg.de/) (Letunic et al., 2006; Haider et al., 2023a) and NCBI CDD (Conserved Domain Database) (https://www.ncbi.nlm.nih.gov/Structure/cdd/cdd.shtml) (Marchler-Bauer et al., 2013) with the default parameters.

### 2.2 Data retrieval of *NPR1*-like genes

The NPR reference sequences from *A. thaliana* were employed to identify homologous genes in the protein database of 11 citrus species via the https://www.citrusgenomedb.org/blast platform, with an E-value threshold of < 1.2 × 10^−14^. Subsequently, genomic, protein, CDS, and promoter sequences for all NPR genes were retrieved. In-depth analysis included the thorough examination of protein sequences to pinpoint representative sequences within duplicated genes. This process involved the utilization of TBtools and the verification of the existence of distinctive NPR domains, encompassing ANK repeats and N-terminal BTB/POZ domains. Additionally, motif analysis was conducted on these protein sequences using the motif finder tool (https://www.genome.jp/tools-bin/search_motif_lib) (Arabbeigi et al., 2018). The identified motifs were then compared against the canonical pattern found in “*AtNPR1*”-like proteins.

### 2.3 Gene characterization and structure analysis of *NPR1*-like genes

Multiple sequence alignments of *CsNPRs*, *CrNPRs*, *CfNPRs*, *CcNPRs*, *ClNPRs*, *CmdNPRs*, *CmNPRs*, *AbNPRs*, *PtNPRs*, and *CiNPRs* were generated using the MUSCLE Method in the Mega-X program and were visualized (Xu et al., 2020). Gene Structure Display Server (GSDS, http://gsds.cbi.pku.edu.cn/) (Guo et al., 2007) exhibited the exon–intron structures of *NPR1*-like genes in different citrus species. Moreover, NCBI-Conserved Domain Database (CDD) was used for the identification of the conservative domain compositions of 63 distinct *CsNPRs*, *CrNPRs*, *CfNPRs*, *ClNPRs*, *CcNPRs*, *CmdNPRs*, *CmNPRs*, *AbNPRs*, *PtNPRs*, and *CiNPRs* and the results of which were visualized by the TBtools software (https://www.genome.jp/tools/motif/) (Nakasu et al., 2019). The MEME Suite, accessible at https://meme-suite.org/meme/tools/meme, was utilized to identify protein motifs encoded by these genes. Additionally, an online tool was employed to pinpoint the locations of these motifs within the peptide sequences (Haider et al., 2023b).

### 2.4 Phylogeny analysis

Furthermore, the protein sequences of *A. thaliana* were retrieved from the GenBank (Sayers et al., 2022) database (https://www.ncbi.nlm.nih.gov/genbank/) to find out the phylogenetic integration among these *NPR1-*like proteins and CsNPRs, CrNPRs, CfNPRs, CcNPRs, ClNPRs, CmdNPRs, CmNPRs, AbNPRs, PtNPRs, and CiNPRs-like proteins. The full-length sequences of *Arabidopsis* proteins and the protein sequences of *NPR1*-like genes in 10 different citrus species were aligned using the Clustal W method (Nakasu et al., 2019). As a result, an unrooted phylogenetic tree was generated using the maximum-likelihood (ML) algorithm, employing MEGA-X software, with the specified parameters including the bootstrap method (1,000 replicates), the Jones–Taylor–Thornton model, and the inclusion of all sites (Shafiq et al., 2024).

### 2.5 Nonsynonymous (Ka) and synonymous (Ks) substitution rates calculation

The Ks and Ka values of genes from *CsNPRs*, *CrNPRs*, *CfNPRs*, *CcNPRs*, *ClNPRs*, *CmdNPRs*, *CmNPRs*, *AbNPRs*, *PtNPRs*, and *CiNPRs* were also determined. To achieve this, duplicate gene pairs resembling *NPR1*-like genes, originating from various duplication mechanisms, were employed to calculate substitution rates for Ka and Ks. TBtools were utilized to compute Ka and Ks values for these duplicated gene pairs and their respective Ka/Ks ratios using the simple Ka/Ks calculator feature. The Ka/Ks ratio was assessed to elucidate the molecular evolutionary rates of each gene pair. Generally, a Ka/Ks value <1 suggests purifying selection in evolution, Ka/Ks equals to 1 indicates neutral selection, while Ka/Ks >1 signifies positive selection (Haider et al., 2023b). Furthermore, the calculation of the divergence time for these gene pairs was conducted using the formula t = Ks/2r, with r (1.5 × 10^−8^) serving as the representative value for neutral substitution rates (Campos et al., 2023).

### 2.6 Subcellular localization analysis

Subcellular localization of identified genes expressed was performed by using the WoLF PSORT program (https://wolfpsort.hgc.jp/) (Horton et al., 2006). Similarly, other factors regarding *CsNPRs*, *CrNPRs*, *CfNPRs*, *CcNPRs*, *ClNPRs*, *CmdNPRs*, *CmNPRs*, *AbNPRs*, *PtNPRs*, and *CiNPRs* genes and their respective coded proteins like molecular weight (Mw), isoelectric point (pI) value, amino acid (AA) length, length of mRNA, and chromosome number of all *CNPR* genes were gathered from the online tools like ExPASy-Protparam Tool (https://web.expasy.org/protparam/) (da Silva et al., 2020) and Citrus genome database (https://www.citrusgenomedb.org/).

### 2.7 Chromosomal mapping of NPR genes

The chromosomal Id, start and end position of *NPR1* genes of *CsNPRs*, *CrNPRs*, *CfNPRs*, *CcNPRs*, *ClNPRs*, *CmdNPRs*, *CmNPRs*, *CbNPRs*, *PtNPRs*, and *CiNPRs* were retrieved using “Citrus Genome Database” (https://www.citrusgenomedb.org), and the length of chromosomes was found using “FASTA stats” tool (https://bio.tools/gfastats). Three files, “Chromosome ID and length,” “Chromosome ID, start position/end position,” and “gene pairs”, derived from the Ks/Ka analysis, were driven to the “Gene Location Visualize Advance” window of TBtools, and the graph of chromosomal mapping was retrieved (Islam et al., 2023).

### 2.8 Cis-regulatory element and synteny data retrieval

PlantCARE database (http://bioinformatics.pAb.ugent.be/webtools/plantcare/htmL/) (Haider et al., 2023b) was used to identify Cis-regulatory elements. The promoter sequences of *NPR1*-like genes were extracted from genome sequences of 10 citrus species by TBtools software. Chromosome IDs and coordinates were sourced from the Citrus Genome Database, then Advanced Circos in TBtools was used to create the synteny graph.

### 2.9 Citrus NPR gene expression in diverse tissues and organs

The study of ESTs (expressed sequence tags) to find resistance genes in citrus species and characterize their expression levels across multiple citrus plant tissues and organs, leveraging the online NCBI Gene Expression Omnibus (GEO) database (Grechkin et al., 2017). The Blast P search was performed using a default parameter with an e-value of 1e−04. Various genes exhibited expression in distinct segments of the citrus plant, encompassing the flower, root, fruit, leaf, stem, and more, in response to diverse forms of biotic stress and various influencing factors. Heat maps were used to analyze the expression patterns of specific resistance genes across different tissues and organs in different citrus species.

### 2.10 Plant material

The four citrus cultivars *C. sinensis* (sweet orange), *C. reticulata* (mandarin), *C. limon* (lemon), and *C. fortunella* (kumquat) were inoculated with citrus bacterial culture (*Xanthomonas axonopodis* pv. *citri*) and chosen for gene expression. The experiment was conducted in a greenhouse (day/night temperature, 25°C; light/dark periods, 16/8) using the method of completely randomized design (CRD) and three replicates of each treatment.

### 2.11 RNA extraction and qRT-PCR analysis

mRNA was extracted using the triazole method (Fujino et al., 2016), and first-strand cDNA was synthesized using AB script II cDNA first-strand synthesis kit. SYBR Green (Wiz pure) was used to perform qRT-PCR. Standard curves were created to estimate the proportion of citrus *NPR1*-like genes, and housekeeping gene quantity in samples was determined using real-time PCR (Illumina) and software (https://biomolecularsystems.com/mic-qpcr/software/). To analyze the dynamics of expression, we conducted three biological and three technical replicates. Significance differences were assessed using Student’s t-tests (p < 0.05).

### 2.12 Statistical analysis

The data were subjected to statistical analysis using a two-way factorial completely randomized design (CRD). Analysis of variance (ANOVA) was utilized to assess variances, and means were differentiated using the least significant difference (LSD) method, with a significance level set at p < 0.05. The entire statistical analysis was carried out using the Statistix 8.1 software suite.

## 3 Results

### 3.1 Identification, description, and evolutionary analysis of *NPR1* homologues in citrus varieties

**Figure 1:**
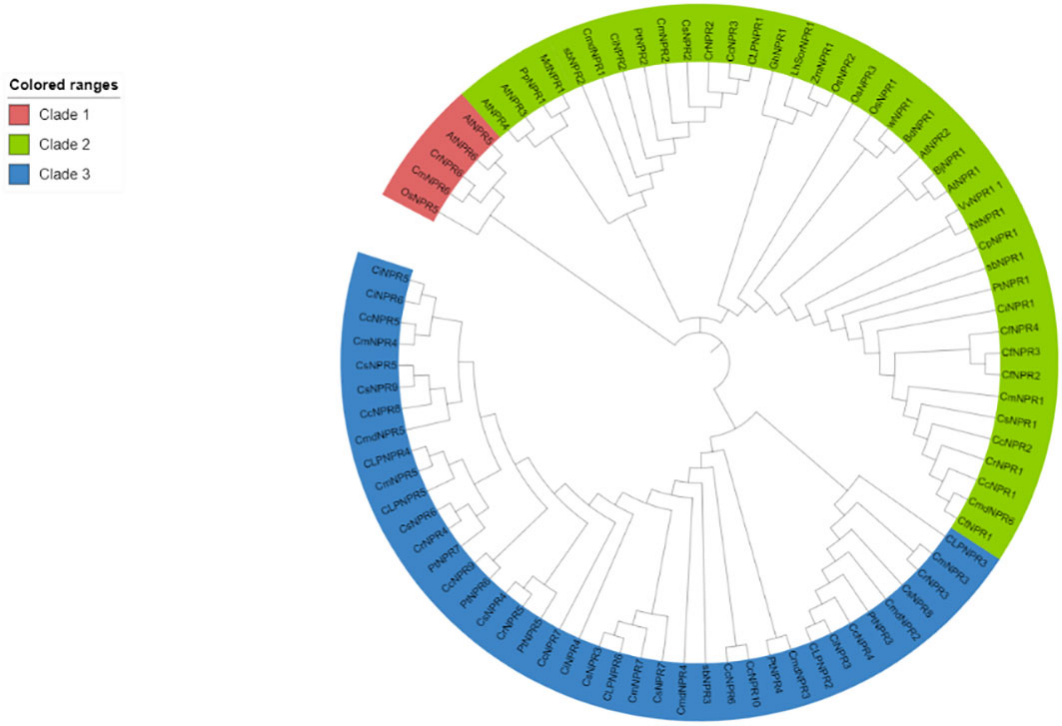
Polygenetic tree of NPR1 like proteins in *CsNPRs, CrNPRs, CfNPRs, CcNPRs, CmdNPRs, CmNPRs, sbNPRs, PtNPRs* and *CiNPRs* and its homologous in, A. thaliana (At).

Approximately 63 non-redundant *NPR1*-like genes were retrieved from the genome of *C. sinensis*, *C. reticulata*, *C. fortunella*, *C. limon*, *C. maxima*, *C. ichangenesis*, *C. maxima*, *P. trifoliata*, *C. climentina*, *A. buxifolia*, and *C. medica*. Utilizing bioinformatics tools like BLAST search, 63 *NPR1*-like genes were discovered in the genome and named as *CsNPRs*, *CrNPRs*, *CfNPRs*, *ClNPRs*, *CcNPRs*, *CmdNPRs*, *CmNPRs*, *AbNPRs*, *PtNPRs*, and *CiNPRs*. Using NCBI CDD Domain analysis, all the proteins that lack N-terminal BTB/POZ domain or ANK repeats in the middle region were excluded. As a result, different species have different number of *NPR1*-like genes, like nine in *C. sinensis*; seven in *C. reticulata*; two in *C. fortunella*; six in *C. limon*, *A. thaliana*, and *C. medica*; 10 in *C. climentina* and *C. ichangensis*; seven in *C. maxima* and *P. trifoliata*; and three in *A. boxifolia* (**Figure 1**).

The mRNA lengths of these CsNPRs, CrNPRs, CfNPRs, CcNPRs, CmdNPRs, CmNPRs, AbNPRs, PtNPRs, and CiNPRs genes have nucleotides that range from 1,119 to 69,428, while the amino acid lengths of their respective proteins vary between 372 and 1,379 amino acids. Additionally, the overall pI values vary from 4.91 to 8.74, and the molecular weight of described genes CsNPRs, CrNPRs, ClNPRs, CfNPRs, CcNPRs, CmdNPRs, CmNPRs, AbNPRs, PtNPRs, and CiNPRs proteins vary from 118,053.42 to 46,773.06 (**Table 1**).

**Table 1:** Physiochemical properties of NPR1 gene family in different citrus species.

	Gene	Accession Number	A. A length	mRNA length	M. W	pI
*C. reticulata*	CrNPR1	MSYJ077460_1	585	4206	65789.71	6.31
	CrNPR2	MSYJ131960_1	587	3267	65938.7	5.7
	CrNPR3	MSYJ281930_1	592	9064	65764.77	5.9
	CrNPR4	MSYJ281950_1	565	69428	63352.37	5.8
	CrNPR5	MSYJ281960_1	800	20268	89049.19	7.45
	CrNPR6	MSYJ281940_1	416	2238	47346.89	8.74
	CrNPR7	MSYJ287170_1	448	2671	49421.35	6.28
*C. sinensis*	CsNPR1	Cs4g14600_1	585	2219	65789.71	6.31
	CsNPR2	Cs2g10790_1	532	2263	65884.67	5.77
	CsNPR3	Cs7g18670_1	576	1732	64992.27	5.64
	CsNPR4	Cs7g18690.1	1045	3203	118053.42	7.44
	CsNPR5	Cs7g18600_1	488	2360	54861.44	6.35
	CsNPR6	Cs7g18670_1	576	1732	64992.27	5.64
	CsNPR7	Cs7g18690_1	1045	3203	118053.42	7.44
	CsNPR8	Cs7g18620_1	1379	4506	155423.04	5.83
	CsNPR9	Cs7g18630_1	577	2056	64773.85	5.65
	CsNPR10	Cs7g18680_1	590	2188	66226.62	5.48
	CsNPR12	orange1_1t02156.1	416	1251	46773.06	6.98
*C. fortunella*	CfNPR1	sjg283810_1	451	4547	50546.84	5.91
	CfNPR2	sjg245720_1	441	4842	48518.38	6.22
*C. limon*	CLNPR1	CL2G008891011.t1_pri	615	1848	69287.44	5.72
	CLNPR2	CL7G026056011.t1_pri	640	1923	71573.98	6.6
	CLNPR3	CL7G026057011.t1_pri	583	1785	65056.09	5.98
	CLNPR4	CL7G026062011.t1_pri	989	2970	110582.54	8.32
	CLNPR5	CL7G026072011.t1_pri	566	1731	63864.33	5.46
	CLNPR6	CL7G026061011.t1_pri	550	1668	61634.47	6.02
*C. clementina*	CcNPR1	clementine0.9_006073m	574	2040	64590.19	6.31
	CcNPR2	clementine0.9_005813m	585	2073	65789.71	6.31
	CcNPR3	clementine0.9_007280m	527	1802	59573.35	5.49
	CcNPR4	clementine0.9_005587m	595	2503	66349.57	6.08
	CcNPR5	clementine0.9_005201m	616	1867	68892.48	5.27
	CcNPR6	clementine0.9_034478m	553	1662	61768.05	5.02
	CcNPR7	clementine0.9_035825m	416	1248	46699.2	5.25
	CcNPR8	clementine0.9_005876m	582	1749	65152.2	5.67
	CcNPR9	clementine0.9_007701m	514	1545	57551.7	5.7
	CcNPR10	clementine0.9_011886m	405	1387	45454.63	4.91
*C. maxima*	CmNPR1	Cg4g008720.1	517	1722	58000.45	5.97
	CmNPR2	Cg2g038060.1	587	2547	65885.66	5.7
	CmNPR3	Cg7g012270.2.1	600	2595	66636.75	6.15
	CmNPR4	Cg7g012290.1	794	2385	89459.31	6.02
	CmNPR5	Cg7g012280.1	593	1782	66655.55	5.55
	CmNPR6	Cg9g017270.1	506	1704	55418.89	6.15
	CmNPR7	Cg7g012320.1	398	1197	43695.7	6.1
*C. ichangensis*	CiNPR1	Ci293640.2	434	3617	48430.58	5.9
	CiNPR2	Ci150300.1	587	2567	66020.83	5.83
	CiNPR3	Ci132570.2	595	2600	66397.57	6.08
	CiNPR4	Ci132560.1	484	1455	54036.56	5.34
	CiNPR5	Ci132530.1	477	1434	52840.71	5.05
	CiNPR6	Ci296910.1	580	1743	64783.49	5.5
*P. trifoliate*	PtNPR1	Ptrif.0001s0987.3.v1.3.1	435	4266	48742.99	5.85
	PtNPR2	Ptrif.0002s2330.2.v1.3.1	587	4440	65916.64	5.91
	PtNPR3	Ptrif.0004s1251.1.v1.3.1	594	4436	66333.51	6.08
	PtNPR4	Ptrif.0004s1256.1.v1.3.1	467	2810	52059.48	5.47
	PtNPR5	Ptrif.0004s1259.1.v1.3.1	407	1875	45680.06	5.59
	PtNPR6	Ptrif.0004s1254.1.v1.3.1	594	4854	66690.75	5.3
	PtNPR7	Ptrif.0004s1252.2.v1.3.1	252	1922	28077.37	5.28
*S. buxifolia*,	SbNPR1	sb23887.4	434	4017	48442.57	5.91
	SbNPR2	sb12905.3	447	2834	50436.9	5.66
	SbNPR3	sb24964.1	445	1337	49597.08	6.26
*C. medica*	CmdNPR1	Cm196830.1	587	2836	65986.78	5.7
	CmdNPR2	Cm144560.1	957	3067	106809.87	5.68
	CmdNPR3	Cm144640.1	372	1119	41541.5	5.67
	CmdNPR4	Cm144610.1	433	1302	48576.58	5.2
	CmdNPR5	Cm144680.1	585	1758	65371.25	5.19
	CmdNPR6	Cm127070.1	398	1197	45000.1	6.09

AA, amino acid sequence length; MW, molecular weight; pI, isoelectric point

The evolutionary tree from the protein sequences of *NPR1*-like was constructed among the various species of citrus (*C. sinensis*, *C. reticulata*, *C. fortunella*, *C. maxima*, *C. medica*, *C. ichangenesis*, *C. limon*), and *A. buxifolia* and *P. trifoliata*, and references the *A. thaliana* species as a model (Cao et al., 1997; Spoel et al., 2003; Peng et al., 2021). A 1,000 bootstrap tree was constructed using the UPGMA model in MegaX software. The phylogeny analysis demonstrated the presence of NPR-like proteins categorized into three distinct classes or clades, matching patterns found in other species. Clade I include 41 NPR-related proteins, and clade II comprised 16 NPR-related proteins. Proteins from the same clade appear to be structurally and functionally related (Sami et al., 2024).

### 3.2 Gene duplication analysis

To estimate the approximate gene divergence time, the TBtools program was utilized, and it furnished values for Ks, Ka, and Ka/Ks ratios as specified. The value of synonymous substitutions per synonymous site was denoted by Ks, while the value of non-synonymous substitutions per non-synonymous site was quantified by Ka. The balance between synonymous and non-synonymous mutations can be seen in the Ka/Ks ratio. The evolutionary investigation revealed six gene pairs associated with gene duplication within the NPR family. The first pair included *ClNPR4*/*CmNPR5*, the second pair *CsNPR4*/*CrNPR5*, the third pair *CrNPR3*/*CsNPR8*, the fourth pair *CmNPR6*/*CrNPR6*, the fifth pair *AtNPR6*/*AtNPR5*, and the sixth pair *AbNPR1*/*AtNPR2* (**Table 2**).

**Table 2:** The expression ka/ks represents the ratio of mutations involving synonymous substitutions (ks) to mutations involving non-synonymous substitutions (ka).

Seq-1	Seq-2	ka	Ks	Ka-ks	T(MYA)	Protein homology
CLPNPR4	CmNPR5	0.0685414	0.090403078	0.758175584	30134359.2	23.5
CsNPR4	CrNPR5	0.0795171	0.11444433	0.694810345	38148110.08	92.3
CrNPR3	CsNPR8	1.8985625	1.740752009	1.090656475	580250669.7	98.5
CmNPR6	CrNPR6	9.80E-04	0.015658189	0.062571218	5219396.255	17.6
AtNPR6	AtNPR5	0.0782242	1.098978008	0.071179016	366326002.7	82.9
sbNPR1	AtNPR2	0.3066207	1.641364596	0.186808411	547121531.9	54.6

The gene duplication over selection and evolutionary pressure to paralogous pairings of NPR genes were calculated based on ks and ka values.

A measure that evaluates the selection pressure affecting amino acid substitutions was the Ka/Ks ratio. When the Ka/Ks ratio declines below 1, it denotes an evolutionary process of purifying selection (Parmley and Hurst, 2007). If this ratio was exceeding value 1, it may indicate the probability of positive selection (Biswas and Akey, 2006). The findings showed that the Ka/Ks ratios varied significantly across NPR groups. The Ka/Ks ratios for *ClNPR4/CmNPR5*, *CsNPR4/CrNPR5*, *CmNPR6/CrNPR6*, *AtNPR6/AtNPR5*, and *AbNPR1/AtNPR2* indicate purifying selection, with values below 1, while the Ka/Ks ratio for *CrNPR3/CsNPR8* suggests positive selection, as it exceeds 1, indicating environmental influence (Sami et al., 2023d).

### 3.3 Genes structure and sequence analysis

**Figure 2:**
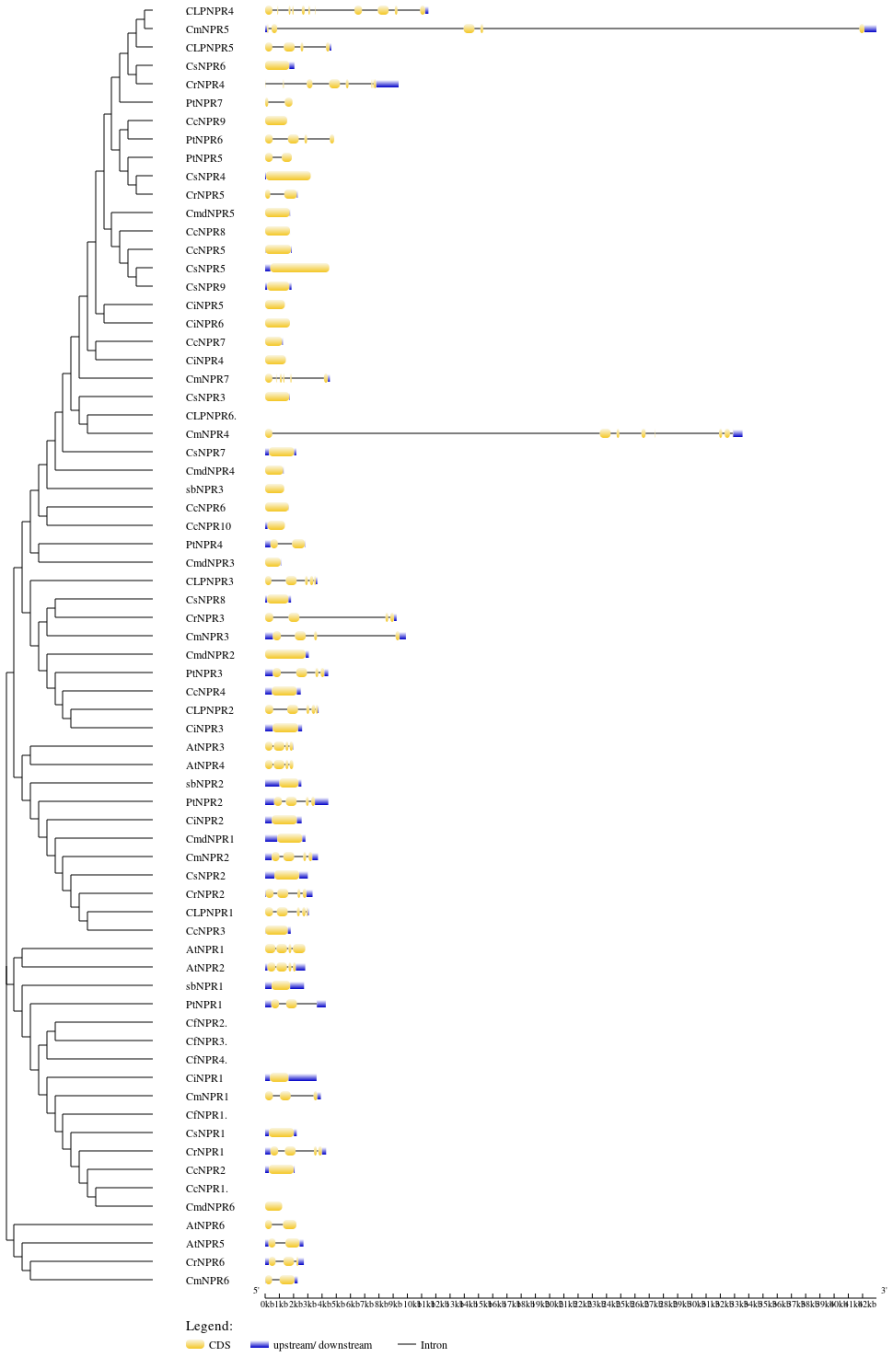
Phylogenetic relationship and gene structure of NPR1 genes from *CsNPRs, CrNPRs, CfNPRs, CcNPRs, CmdNPRs, CmNPRs, sbNPRs, PtNPRs* and *CiNPRs*. The phylogenetic tree was constructed using full length sequences of *CsNPRs, CrNPRs, CfNPRs, CcNPRs, CmdNPRs, CmNPRs, sbNPRs, PtNPRs* and *CiNPRs*-like genes. Yellow boxes indicate exons; and black lines indicate introns.

To gain deeper insights into the functions of *CsNPRs*, *CrNPRs*, *CfNPRs*, *CcNPRs*, *ClNPRs*, *CmdNPRs*, *CmNPRs*, *AbNPRs*, *PtNPRs*, and *CiNPRs* genes, structural features and sequence composition analysis was conducted using online tools GSDS and NCBI-CDD. Intron/exon profiles of NPR genes were generated using GSDS 2.0 (**Figure 2**). Notably, the majority of NPRs, including *CsNPR6*, *CcNPR9*, *CmdNPR5*, *CcNPR8*, *CcNPR5*, *CsNPR5*, *CsNPR9*, *CiNPR5*, *CiNPR6*, *CiNPR7*, *CiNPR4*, *CsNPR3*, *CsNPR7*, *CmdNPR4*, *AbNPR3*, *CcNPR6*, *CcNPR10*, *CmdNPR3*, *CsNPR8*, *CmdNPR2*, *CcNPR4*, *CiNPR3*, *AbNPR2*, *CiNPR2*, *CmdNPR1*, *CsNPR2*, *CcNPR3*, *AbNPR1*, *CiNPR1*, *CsNPR1*, *CiNPR2*, and *CmdNPR6*, exhibited lack of introns. In contrast, the four *Arabidopsis* sequences, *AtNPR1*, *AtNPR2*, *AtNPR3*, and *AtNPR4*, contained two introns, while *AtNPR5* and *AtNPR6* had only one intron each. *ClNPR4* had seven introns; *CmNPR4* had six introns; *CmNPR5*, *CrNPR4*, and *ClNPR3* had four introns; and *ClNPR5*, *PtNPR6*, *CmNPR7*, *CmNPR3*, *PtNPR3*, *ClNPR3*, *AtNPR3*, *AtNPR4*, *PtNPR2*, *CmNPR2*, and *CrNPR1* had three introns. *CrNPR6*, *CmNPR1*, and *CrNPR3* had two introns, while *PtNPR5*, *CmdNPR5*, *PtNPR4*, and *CmNPR6* possessed a single intron only. The number of introns per gene ranged from 0 to 7. Interestingly, no results were obtained for *CcNPR1*, *CfNPR1*, *CfNPR2*, and *CcNPR6*. These findings highlight significant variations in the number of introns–exons among *CsNPRs*, *CrNPRs*, *CfNPRs*, *ClNPRs*, *CcNPRs*, *CmdNPRs*, *CmNPRs*, *AbNPRs*, *PtNPRs*, *CiNPRs*, and *A. thaliana* genes.

### 3.4 Motif analysis

**Figure 3:**
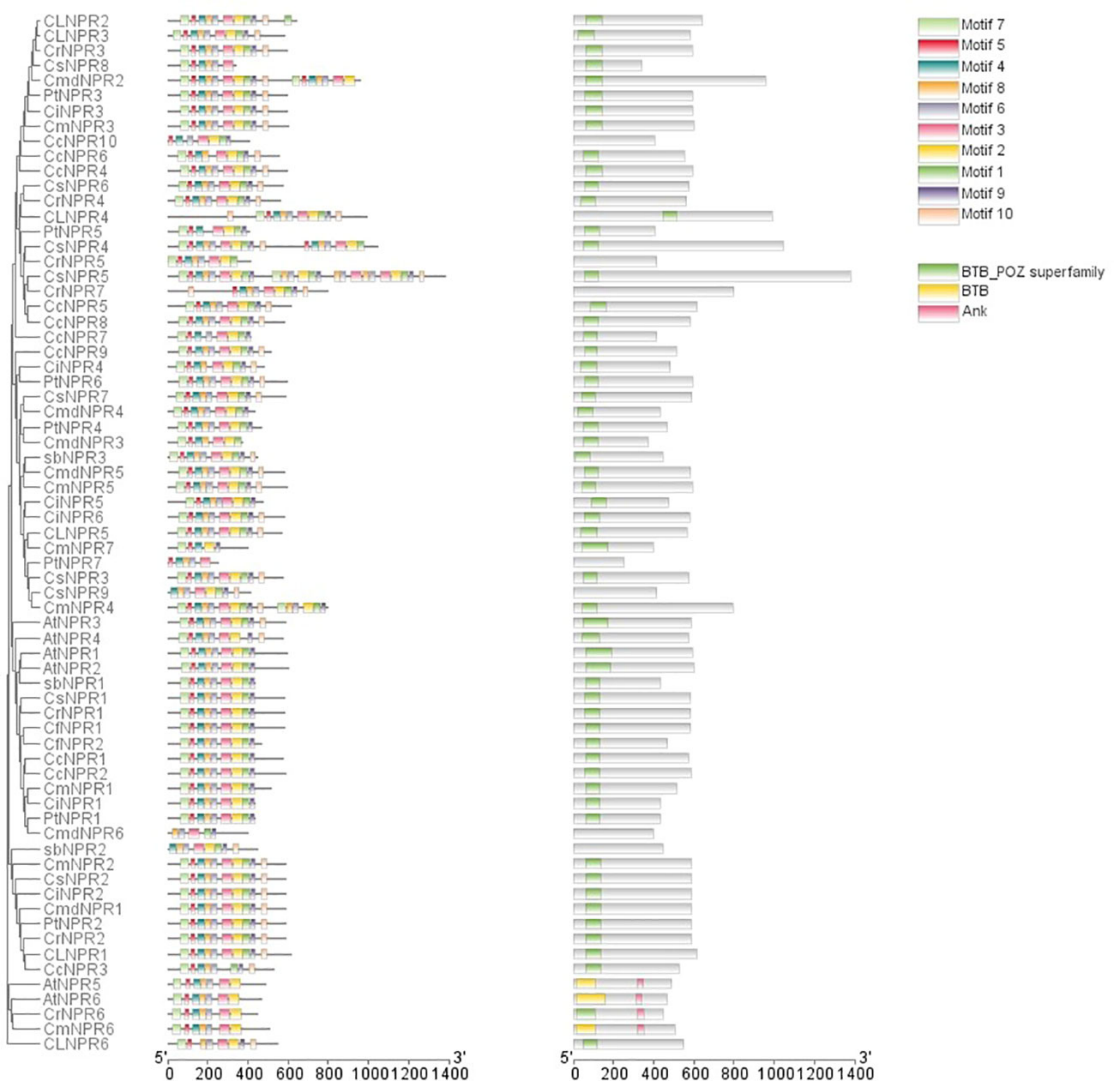
The domain arrangements of putative *NPR1*-like genes in *C. sinensis, C. reticulata, C. fortunella, C. limon, C. medica, C. maxima, C. clementina, C. ichangensis, P. trifoliate, A. buxifolia*, and *A. thaliana* connected with the phylogenetic tree.

Conserved domain analysis was employed to filter out sequences exclusively featuring the BTB domain. Following that, motif analysis, along with conserved domain analysis, was carried out using TBtools. The analysis revealed that several genes, including *ClNPR4*, *CmNPR5*, *ClNPR5*, *CsNPR6*, *CcNPR9*, *PtNPR6*, *CcNPR8*, *CcNPR5*, *CmNPR2*, *CsNPR2*, *CrNPR2*, *CLNPR1*, *AtNPR1*, *AtNPR2*, *AbNPR1*, *PtNPR1*, *CfNPR2*, and *CrNPR4* possessed motifs in the order of 8, 7, 2, 9, 5, 6, 1, 4, 3, and 10. However, *PtNPR5* shows all these motifs except motif 5, while *CrNPR5* featured all of them except for motifs 8 and 10, and *CsNPR4* included motifs 7, 2, 9, 5, 6, 1, 4, 3, and 10, in addition to others. *CsNPR5* and *CsNPR9* displayed motifs 8, 9, 7, 2, 5, 6, 1, 4, 3, and 10, followed by motifs 9, 5, 6, 7, 4, 3, and 10. Some sequences had minor variations, such as *ClNPR6* lacking motifs 2 and 3; *CcNPR6* lacking motif 5; *CmdNPR3* and *CmdNPR2* featuring motifs 7, 2, 9, 5, 6, 1, and 4 in addition; and *CrNPR5* missing motifs 8 and 10, along with motifs 6, 1, 4, 3, and 10. *CsNPR7* possessed an additional motif 10; *CmdNPR6* lacked motifs 8, 7, 2, 1, and 6; *CsNPR8* lacked motifs 4, 1, 3, and 10; *ClNPR2* had an additional motif 3; *CmNPR4* had motifs 8, 9, 1, 4, 3, and 10 in addition; *CmdNPR3* lacked motifs 5, 6, and 10; *AtNPR3* and *AtNPR4* were lacking motif 3; and *CcNPR3* lacked motif 1. *PtNPR7* exhibited motifs 7, 2, 9, 5, 6, and 1, while *CiNPR5* lacked motif 6, as did *CiNPR6* and *CcNPR7*. *CmNPR7* only possessed motifs 8, 7, 2, 9, 4, and 10. *CiNPR1*, *CmNPR1*, *CfNPR1*, *CsNPR1*, *CrNPR1*, *CcNPR2*, and *CcNPR1* did not have motif 6, whereas *AtNPr5*, *AtNPR6*, *CrNPR6*, and *CmNPR6* had an additional motif 6 (**Figure 3**).

### 3.5 Subcellular localization of NPR-like gene

**Figure 4:**
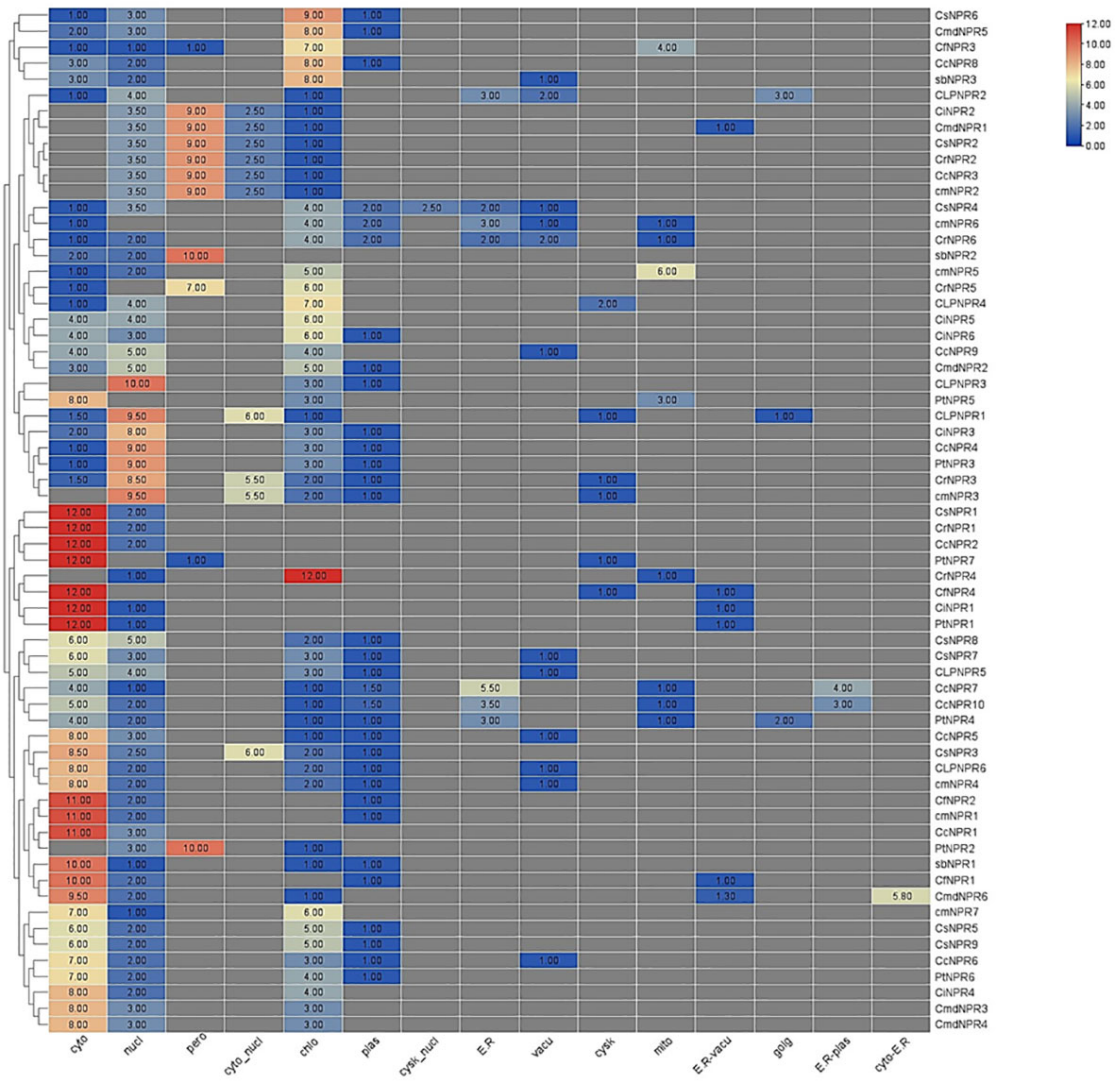
An abundance of putative *NPR1*-like genes in various species of citrus, connected to a phylogeny tree, enables a better understanding of gene function and analogy. Cytoplasm exhibited the highest abundance of *CsNPR1, CrNPR1, CcNPR2, PtNPR7, CiNPR1*, and *PtNPR1*, while various organelles had distinct but lower quantities of these proteins, indicating diverse subcellular distributions.

The subcellular localization analysis indicated that cytoplasm was the predominant location for *CsNPR1*, *CrNPR1*, *CcNPR2*, *PtNPR7*, *CiNPR1*, and *PtNPR1*. Chloroplasts and plastids showed lower quantities of *CsNPR9*, *CsNPR7*, *CLNPR5*, *CcNPR7*, *CcNPR10*, *PtNPR4*, *CcNPR5*, *CsNPR3*, *CLNPR5*, *CmNPR4*, *CfNPR2*, *CmNPR1*, *CcNPR1*, *PtNPR2*, *AbNPR1*, *CfNPR1*, *CmdNPR6*, *CmNPR7*, *CsNPR5*, *CsNPR9*, *CcNPR6*, *PtNPR6*, *CiNPR4*, *CmdNPR3*, and *CmdNPR4*. Chloroplasts had lower levels of *ClNPR1*. Cyto-nucleus contained reduced amounts of *ClNPR1*, *CiNPR3*, *CcNPR4*, *PtNPR3*, *CrNPR3*, *CcNPR3*, and *CmdNPR2*. Peroxisomes contained minimal quantities of *CiNPR2*, *CmdNPR1*, *CsNPR2*, *CrNPR2*, and *CcNPR3*. The nucleus was the primary site for *ClNPR3*, *ClNPR1*, *CiNPR3*, *CcNPR4*, *PtNPR3*, *CrNPR3*, and *CmNPR3*, with *CmNPR6*, *CrNPR5*, and *PtNPR7*, absent from the nucleus. Other proteins were present in lower amounts in the nucleus. ER exhibited limited concentrations of *CcNPR7*, *CcNPR10*, *PtNPR4*, *CsNPR4*, *CmNPR6*, *CrNPR6*, *CsNPR6*, *CmdNPR5*, and *CcNPR8*. Vacuoles contained *CcNPR6*, *CmNPR4*, *CLNPR6*, *CcNPR5*, *CLNPR5*, *CsNPR7*, *CCNPR9*, *CrNPR6*, *CmNPR6*, *CsNPR4*, *CLNPR2*, and *AbNPR3* in small quantities. Mitochondria housed the highest quantity of *CmNPR5*, while *CcNPR7*, *CcNPR10*, *PtNPR4*, *CrNPR4*, *PtNPR5*, *CrNPR6*, and *CmNPR6* were present in smaller amounts. R-vacuoles contained small numbers of *CmdNPR6*, *CfNPR1*, *PtNPR1*, *CiNPR1*, and *CmdNPR1*. Golgi apparatus had *ClNPR2*, CL*NPR1*, and *PtNPR4* in small quantities. ER plastids contained *CcNPR10* and *CcNPR7* in lower numbers, and cyto-ER contained only *CmdNPr6* in moderate quantities (**Figure 4**).

### 3.6 Chromosomal mapping of NPR genes

**Figure 5:**
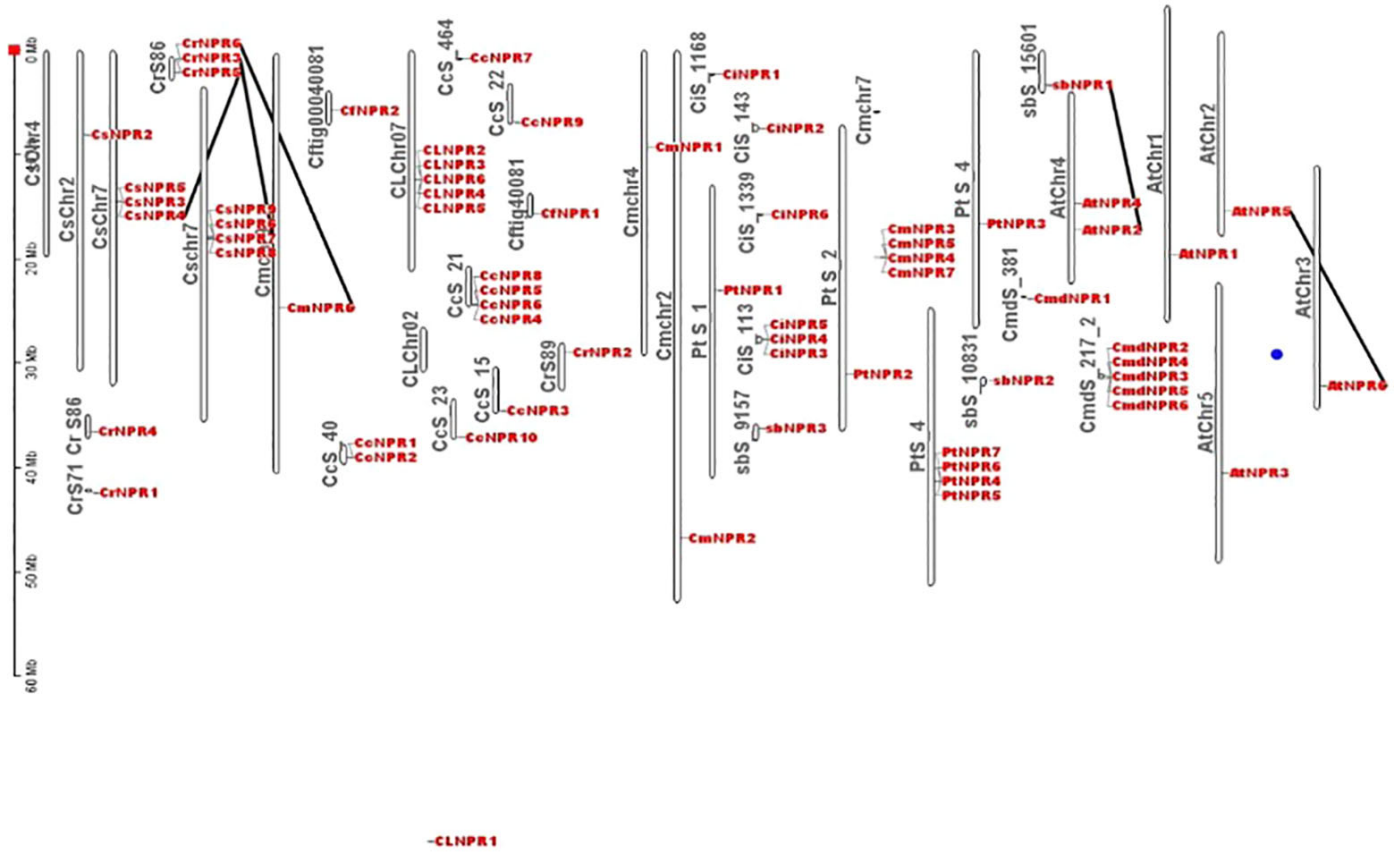
Different citrus species chromosomes location and duplication occurrences to *NPR1*-like genes revealed 5 orthologous pairs, e.g., *CrNPR6* in *C. reticulata* aligns with *CsNPR4* in *C. sinensis*. Additionally, *AbNPR1* in A. buxifolia corresponds to *AtNPR2* in *A. thaliana*, while *AtNPR5* aligns with AtNPR6 within *A. thaliana*.

Comparative chromosomal mapping of several citrus species, including *C. sinensis*, *C. climentina*, *C. medica*, *C. maxima*, *C. ichangenesis*, *C. limon*, *C. fortunella*, *A. buxifolia*, and *P. trifoliata*, about *A. thaliana*, revealed the presence of five pairs of orthologous genes among these citrus species. Specifically, Scaffold 86 of *C. reticulata* contained *CrNPR6*, which had an ortholog, *CsNPR4*, on chromosome 7 of *C. sinensis*. Additionally, *CrNPR3* on Scaffold 86 of *C. reticulata* exhibited orthology with *CsNPR7* on chromosome 7 of *C. sinensis*, while *CrNPR5* on Scaffold 86 of *C. reticulata* shared orthology with *CmNPR6* on chromosome 9 of *C. maxima*. Furthermore, *AbNPR1* on Scaffold 15601 of *A. buxifolia* was found to be orthologous to *AtNPR2* on chromosome 4 of *A. thaliana*, and *AtNPR5* on Chromosome 2 of *A. thaliana* displayed orthologous relationships with *AtNPR6* on chromosome 3 of *A. thaliana* (**Figure 5**).

### 3.7 Cis-regulatory elements in *NPR1*-like genes

**Figure 6:**
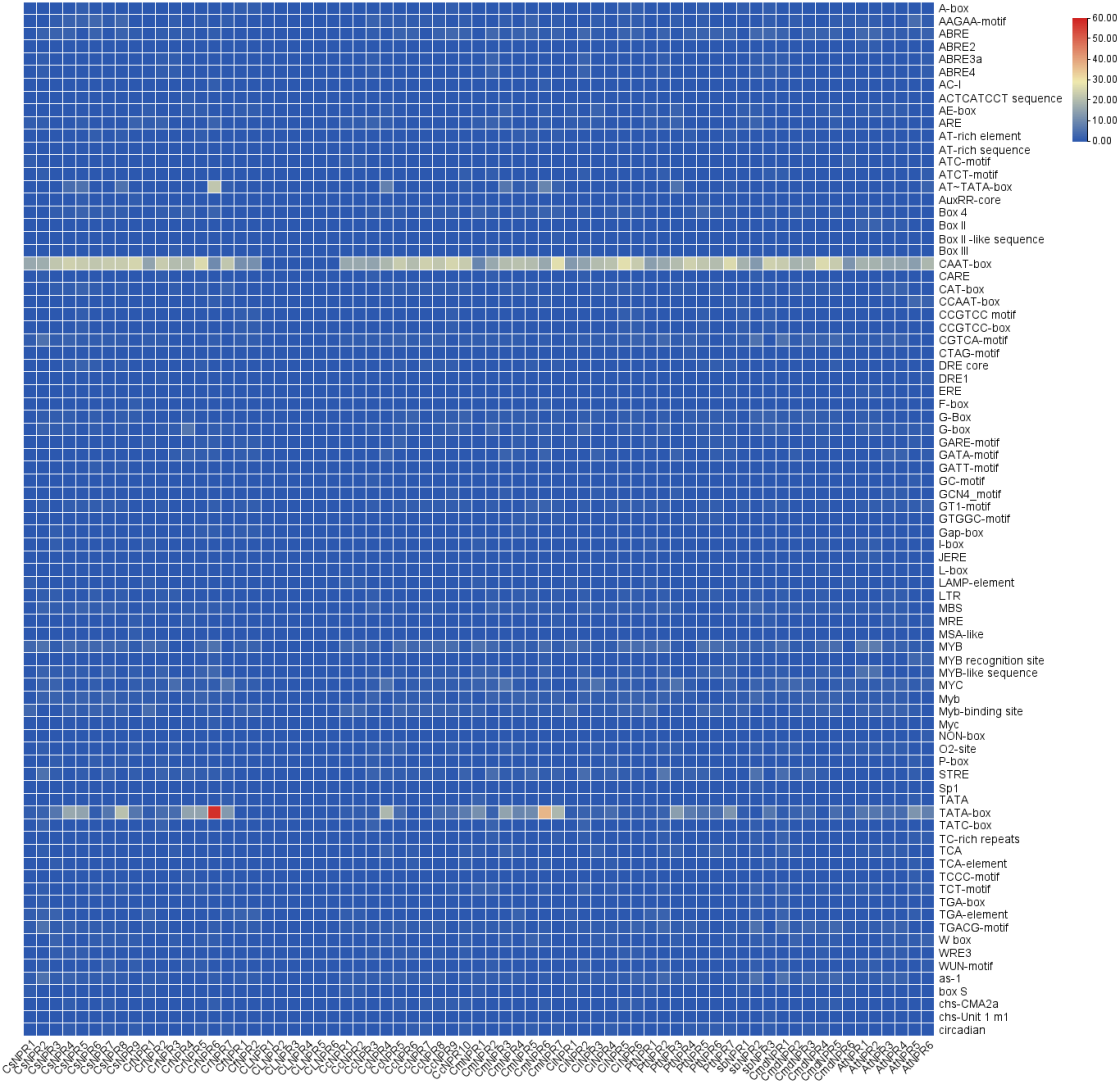
Illustration of cis elements of NPR1 in different citrus spiecies.

Numerous cis-regulatory elements were identified within the 55 promoter regions of *CsNPRs*, *CrNPRs*, *CfNPRs*, *CcNPRs*, *ClNPRs*, *CmdNPRs*, *CmNPRs*, *AbNPRs*, *PtNPRs*, and *CiNPRs*, including widely observed CAAT and TATA boxes. Additionally, phytohormone-responsive elements such as ABRE, TGACG-motif, TCA-element, P-box, GARE-motif, TATC-box, TGA-box, and AuxRR-core were detected across various genes and are involved in the response to auxin, GA, SA, abscisic acid, etc., prominently in *CsNPRs*, *CrNPRs*, *CcNPRs*, *CfNPRs*, *CiNPRs*, *CmdNPRs*, *CmNPRs*, *PtNPRs*, *AbNPRs*, and *AtNPRs*. Light-responsive elements such as Box 4, CAG-motif, G-Box, GT1-motif, ACE, AT1-motif, TCT-motif, I-box, TCCC-motif, chs-CMA2a, AE-box, ATCT-motif, GATA-motif, Gap-box, LAMP-element, GA-motif, Pc-CMA2c, and Sp1 were notably present in various genes. Furthermore, specific cis-regulatory elements like LRR, ACII, and ref2f-1 were discovered in selected genes, implicated in regulating circadian rhythm, zein metabolism, anaerobic induction, and drought stress among other processes (**Figure 6**).

### 3.8 Syntenic relationship of NPR gene of different plants

**Figure 7:**
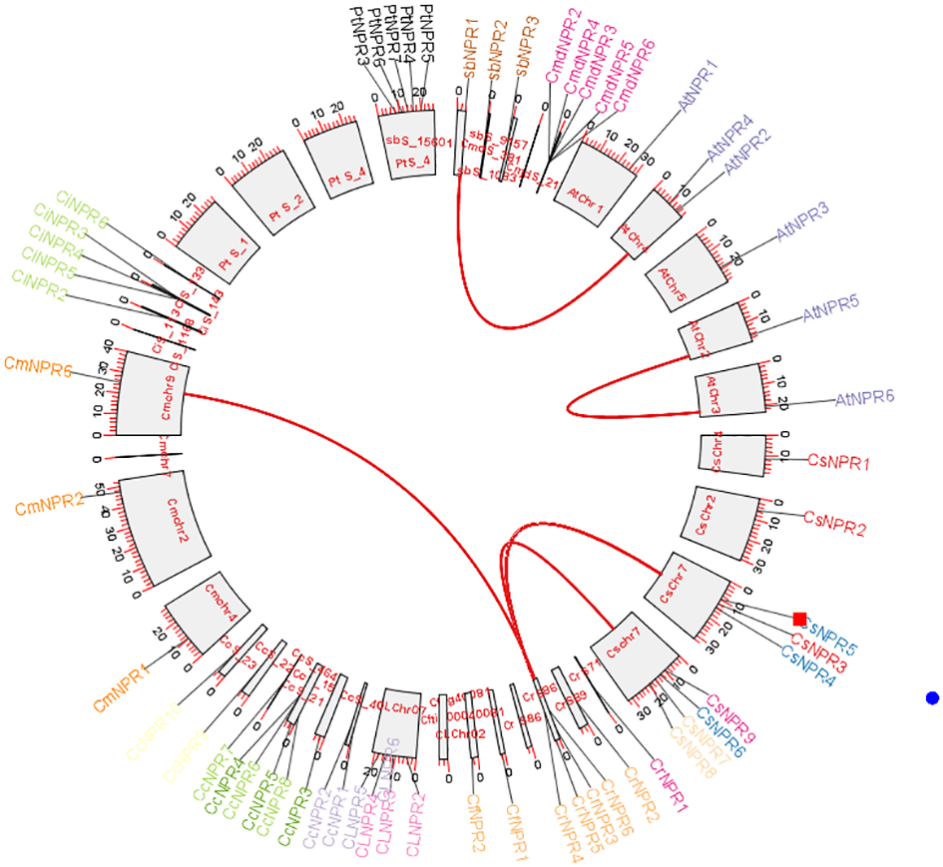
A Circos plot depicting the *NPR1*-like gene family in citrus species. The red connections indicate instances of gene duplications occurring both within and between various species. Synteny analysis revealed collinearity relationships among orthologs from various citrus species and identified specific gene matches, such as *AbNPR1* in *A. buxifolia, AtNPR5* in *A. thaliana*, and *CrNPR3, CrNPR4, CrNPR5, CrNPR6* in *C. reticulata* with their respective orthologs in related species.

To determine the collinearity relationships among orthologous genes of *C. sinensis*, *C. fortunella*, *C. reticulata*, *C. medica*, *C. maxima*, *C. limon*, *A. buxifolia*, *P. trifoliata*, *C. ichangenesis*, and *C. climentina*, a synteny analysis was conducted. Notably, scaffolds 1, 2, and 4 of *P. trifoliata* did not exhibit orthologous gene counterparts. In *A. buxifolia*, *AbNPR1* genes located on scaffold 10 shared orthologs with chromosome 4 of *A. thaliana*, which also houses *AtNPR4* and *AtNPR3*. Similarly, in *A. thaliana*, the gene *AtNPR5* found on chromosome 2 exhibited orthologs on chromosome 3, including AtNPR6. Moreover, genes located on scaffold 86 of *C. reticulata*—specifically, *CrNPR3*, *CrNPR4*, *CrNPR5*, and *CrNPR6* showed orthology with *CmNPR6* on chromosome 9 of *C. maxima* and with genes *CsNPR3* through *CsNPR9* on chromosome 7 of *C. sinensis* (**Figure 7**).

### 3.9 Expression analysis of NPR genes in citrus

*CmNPR1* and *CmNPR5* were derived from previously generated *in silico* data that focused on *NPR1* expression during the flavonoid biosynthesis process in both red and white cultivars *of C. maxima.* The results did not indicate any substantial upregulation or downregulation of *CmNPR1* or *CmNPR5*, implying that *NPR1* might not have a significant influence on flavonoid biosynthesis (Zhao et al., 2020) within *C. maxima*.

#### 3.9.1 Sweet orange fruit expression data across developmental stages

The role of *CsNPR1* in the developmental phases of *C. sinensis* was scrutinized, revealing its active involvement across various stages. Notably, the expression of *CsNPR1* remained consistent across all four treatments, suggesting a stable pattern without significant fluctuations in up- or downregulation. This constancy in expression implies that *CsNPR1* maintains a steady involvement throughout the developmental processes, indicating a potential regulatory role that remains relatively unaltered across the conditions tested. The observed consistency in expression underscores the significance of *CsNPR1* in the intricate orchestration of developmental events within *C. sinensis*.

#### 3.9.2 RNA-seq and targeted metabolomics reveal *C. sinensis* leaf responses to prolonged low pH exposure

In the face of diverse low pH conditions ranging from 2 to 6, tested at intervals of 1 h, 2 h, 3 h, 4 h, and 10 h, *CsNPR1* displayed a remarkable resilience. Importantly, throughout these varying acidic stress conditions, there was no discernible alteration in the expression of *CsNPR1*. This steadfastness in expression levels strongly suggests that *CsNPR1* remains unaffected by acidic stress within the tested pH range and exposure durations. The consistent expression of *CsNPR1* despite the challenging acidic conditions underscores its robust nature and resilience to environmental stressors, highlighting its potential role as a key player in mitigating the impact of low pH stress on the biological processes it governs *NPR1* (Zhang et al., 2022).

#### 3.9.3 Analyzing cuticle regulation in Newhall navel oranges during fruit development and ripening

*CsNPR1* expression was identified in the regulation of cuticle formation during two distinct developmental stages, a finding consistent across two separate replicates. Importantly, there were no significant fluctuations observed in the expression levels of *CsNPR1* during these stages. This stability in expression implies that *CsNPR1* consistently plays a role in the control of cuticle formation at these specific developmental points. The lack of notable changes in *CsNPR1* expression levels underscores its reliability as a regulator in the intricate processes involved in cuticle development, emphasizing its potential importance in maintaining the integrity and protective function of the cuticle across these specific developmental milestones.

#### 3.9.4 The expression levels of CNPRs genes in different citrus species in response to citrus canker using qRT-PCR

**Figure 8:**
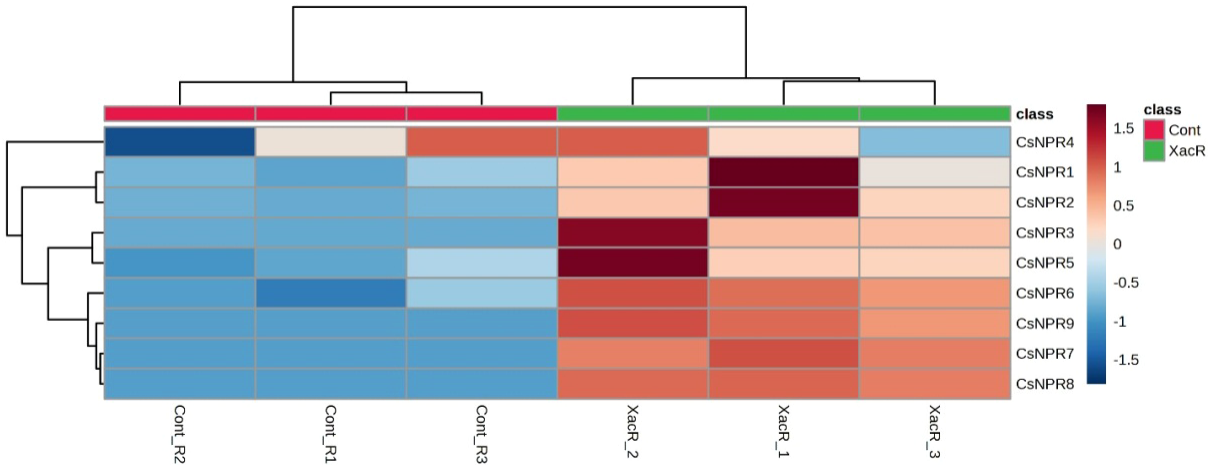
Relative expression of *NPR1* genes in *C. sinensis* infected and healthy plants.

The quantitative analysis using qRT-PCR revealed the presence of *NPR1* genes expression in *C. sinensis* following inoculation with *Xanthomonas axonopodis* pv. *citri*. Out of the nine *CsNPR1* genes, statistical analysis showed that three genes (*CsNPR1*, *CsNPR4*, and *CsNPR5*) did not exhibit significant differences (**Figure 8**). Conversely, the remaining six genes (*CsNPR2*, *CsNPR3*, *CsNPR6*, *CsNPR7*, *CsNPR8*, and *CsNPR9*) displayed statistically significant responses. Notably, all samples that were treated with infected citrus bacterial canker exhibited higher relative expression of the *NPR1* gene compared to the control group. Among them, *CsNPR7*- and *CsNPR9*-treated samples demonstrated the highest gene expression levels. However, it is important to note that control samples displayed higher expression levels than the other control samples.

**Figure 9:**
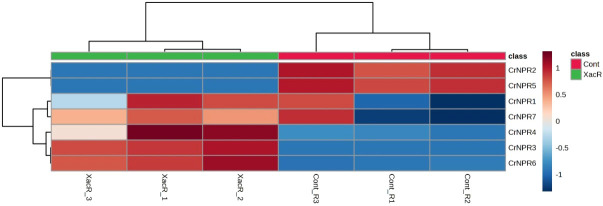
Relative expression of *NPR1* genes in *C. reticulata* infected and healthy plants.

The results obtained from qRT-PCR analysis indicated the expression of *NPR1* genes across all treatments in *C. reticulata*. Notably, all *NPR1* genes in *C. reticulata* displayed statistically significant expression, except for *CrNPR1*, which exhibited varying results. Among these genes, *CrNPR3*, *CrNPR4*, and *CrNPR6* demonstrated the highest levels of expression in the treated samples compared to the control group, while *CrNPR2* and *CrNPR5* exhibited higher expression levels in the control samples than in other genes (**Figure 9**).

**Figure 10:**
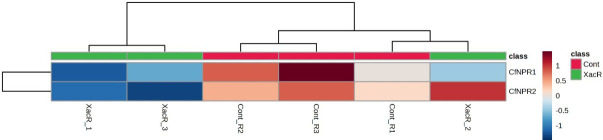
Relative expression of *NPR1* genes in *C. fortunella* infected and healthy plants.

In the case of *C. fortunella*, the real-time qPCR findings showed significant expression of *NPR1* genes, particularly *CfNPR1* and *CfNPR2*. Interestingly, *CfNPR2* displayed increased expression in the control samples compared to the treated (infected with citrus bacterial canker) samples (**Figure 10**).

**Figure 11:**
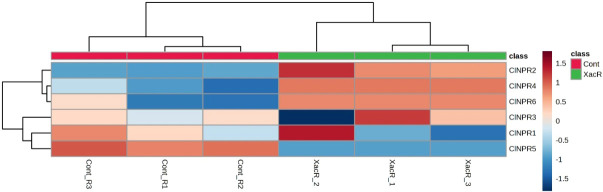
Relative expression of *NPR1* genes in *C. limon*-infected and healthy plants.

Similarly, *NPR1* genes were found to be expressed in all treated samples of *C. limon* compared to the control. Among these, *ClNPR1*, *ClNPR3*, and *ClNPR6* yielded statistically non-significant results, while *ClNPR2*, *ClNPR4*, and *ClNPR5* exhibited statistically significant responses. Notably, *ClNPR2* and *ClNPR4* upregulated the expression of the *NPR1* gene in the treated samples, whereas *ClNPR5* showed higher expression in the control samples compared to the treated ones (**Figure 11**).

## 4 Discussion

This study marks the inaugural genome-wide exploration of *NPR1*-like gene families and their corresponding expression patterns in reaction *to Xanthomonas axonopodis* pv. *citri* infection within four distinct citrus species (Cao et al., 1997; Spoel et al., 2003; Li et al., 2018; Peng et al., 2021).

*NPR1* (Non-expressor of pathogenesis related genes 1) serves as the authentic receptor for salicylic acid, playing a crucial role in plant immunity (Cao et al., 1997; Spoel et al., 2003; Li et al., 2018; Peng et al., 2021). *NPR1* plays a pivotal role in the signaling pathway of salicylic acid and systemic acquired resistance (SAR) in plants. Additionally, other members of the *NPR1* gene family are associated with various reactions to both biotic and abiotic stresses (Cao et al., 1997; Peng et al., 2021). The basic significance of studies on *NPR1* is due to its extensive role in plant defense (Cao et al., 1997; Peng et al., 2021). It is generally postulated that salicylic acid-based defense shields plants against host- or semi-host-dependent pathogens, while jasmonic acid-based defense protects against necrotrophic pathogens (Wally et al., 2009). This study represents the first comprehensive examination of the *NPR1*-like gene family in various citrus species, identifying a total of 63 *NPR1*-like genes across 10 citrus species (Irfan et al., 2023).

The phylogenetic tree illustrates three distinct clades of *NPR* genes, with members within the same clade grouping together based on their presumed similar functions. Gene structure predictions using intron–exon displays and motif analysis revealed that most *NPR1* sequences lacked introns, although some had between 1 and 7. In motif analysis, it was observed that motifs 8, 2, 7, 9, 5, 1, 4, 3, and 10 were commonly found in the majority of sequences. Nevertheless, variations, including the presence or absence of specific motifs, were evident, even among sequences within the same clade. These variations suggest that the *NPR1* gene has undergone structural mutations, including insertions and deletions, over time. The orthologous relationships inferred from gene mapping and syntenic analysis support the notion of a shared evolutionary history among NPR sequences. For *NPR1* to upregulate plant resistance genes, it must localize in the nucleus (Kinkema et al., 2000). Changes in redox potentials influence its nuclear-cytoplasmic localization (Bassene et al., 2011). Typically, *NPR1* exists as a cytoplasmic oligomer under normal conditions. However, upon detecting pathogen infection and salicylic acid accumulation, which alters cellular redox potential, *NPR1* translocates to the nucleus as an active monomer, where it interacts with bZIP transcription factors from the TGA family (Després et al., 2000). The results of subcellular localization confirm that *NPR1* is primarily located in both the cytoplasm and the nucleus, thereby substantiating its involvement in conferring resistance among various citrus species (Spoel et al., 2003; Wally et al., 2009; Després et al., 2000).

Examining selective pressure unveils the benefits of particular amino acid sequence modifications within proteins, offering insights into functional residues and alterations in protein function. The analysis of synteny and chromosomal mapping of *NPR1*-like genes reveals evidence of tandem and segmental duplications, along with the identification of orthologous relationships among different citrus species. This information provides valuable insights into gene duplications, rearrangements, and the evolutionary history of these species. *C. sinensis* and *C. reticulata* share a common ancestor, supported by the presence of various orthologs on chromosome 7 of *C. sinensis* and scaffold 86 of *C. reticulata*. A duplication event occurred in scaffold 86 of *C. reticulata* and chromosome 9 of *C. maxima*, *A. buxifolia*, and *A. thaliana* diverged from a common ancestor, sharing an ortholog.

Analyzing gene expression patterns of *NPR1*-like genes using existing data reveals variations in their expression levels under different stresses (biotic and abiotic).The expression of *CsNPR1* exhibits a remarkable consistency and stability across diverse conditions, reflecting its pivotal role in *C.sinensis* development and stress response. In developmental phases, *CsNPR1* maintains a steady presence, suggesting its regulatory involvement throughout these processes. Its resilience to varying low pH conditions, tested over different time intervals, underscores its robust nature in withstanding acidic stress. Notably, *CsNPR1* expression remains unaffected, implying a key role in mitigating the impact of low pH stress on biological processes. Furthermore, in the regulation of cuticle formation, *CsNPR1* demonstrates reliability with no significant fluctuations in expression levels across distinct developmental stages. Collectively, these findings emphasize the versatility and importance of *CsNPR1*, positioning it as a central player in orchestrating developmental events, responding to environmental stressors, and regulating key processes like cuticle formation in *C. sinensis.* A joint transcriptome analysis was conducted to indicate changes in gene expression of *CmNPR1* and *CmNPR5* during flavonoid biosynthesis, although the differences were not significant (Chen et al., 2022). When *C. sinensis* is subjected to acidic stress for a long time, the expression of *CsNPR1* modulates in the leaves, although this variation is minimal. Similar variations in *CsNPR1* expression were detected during cuticle development in Newhall navel orange fruits (Liu et al., 2022). In *C. sinensis*, all *NPR1-*like genes exhibited upregulated expression in response to Xac infection compared to the control. *CsNPR3*, *CsNPR7*, *CsNPR8*, and *CsNPR9* lacked significant expression in the control group. Except for *CrNPR2* and *CrNPR5*, which exhibited no significant expression in Xac-infected samples, all *NPR1-*like genes in *C. reticulata* showed higher expression. Conversely, *NPR1-*like genes in *C. fortunella* exhibited downregulated expression in response to Xac treatment compared to control samples, while in *C. limon*, *NPR1*-like genes were downregulated except for *ClNPR4* and *ClNPR6*.

Citrus bacterial canker (*Xanthomonas axonopodis* pv. *citri*) stress was studied in terms of *NPR1*-like genes and their associated expression. Citrus species were picked based on their reported resistance to citrus bacterial canker. Two resilient species, *C. sinensis* and *C. limon*, and two vulnerable species, *C. sinensis* and *C. limon*, were picked for a complete comparative study. *C. sinensis* and *C. limon* are vulnerable to citrus bacterial canker, although *C. reticulata* and *C. fortunella* are resistant (Licciardello et al., 2022). The first identified and characterized citrus bacterial canker through cultural, biochemical, and molecular analysis, confirming the presence of *Xanthomonas axonopodis* pv. *citri*, a Gram-negative bacterium (Chaudhari et al., 2022). PCR results using *Xanthomonas axonopodis* pv. *citri*-specific primers XACF (5′-CGTCGCAATACGATTGGAAC-3′) and reverse primer XACR (5′−CGGAGGCATTGTCGAAGGAA-3′) yielded a 581-bp product.

Previous investigations into *NPR1*-like gene families in model organism *A. thaliana* had already established the importance of these families of genes in enhancing tolerance to both biotic and abiotic stress factors (Cao et al., 1997; Després et al., 2000; Kinkema et al., 2000; Spoel et al., 2003; Wally et al., 2009; Peng et al., 2021). This current study unveils alterations in the expression analysis of *NPR1*-like genes in response to an attack by Xac bacteria, with varying responses observed across different citrus species. Notably, in *C. limon* and *C. sinensis*, there was an upregulation in the expression of *NPR1*-like genes. Conversely, in *C. fortunella*, a lower expression of *NPR1*-like genes was observed under Xac attack, rendering it more resistant to the pathogen. *C. reticulata* exhibited varying *NPR1*-like gene expression, indicating a moderate level of resistance to Xac attack. These findings were further corroborated by physiological and biochemical analyses in *C. limon*, which showed decreased levels of chlorophyll a and b, alongside an increase in flavonoid and carotenoid compounds, known for their roles in defense mechanisms against biotic stress. However, the production of secondary metabolites like phenols and soluble proteins was suppressed. Additionally, the activity of vital enzymes, such as catalase and peroxidase, was found to decrease, subsequently impeding the plant’s growth and development. This groundbreaking research addresses a critical gap by conducting the inaugural analysis of the correlation between *NPR1*-associated genes and diverse physiological plant parameters in both citrus cultivars susceptible and resistant to citrus bacterial canker. The findings offer potential implications for the development of disease-resistant citrus varieties through employing multiple breeding techniques, including double haploid technology, hybridization, tissue culture, and backcrossing.

## 5 Conclusion

In conclusion, the comprehensive genome-wide analysis of *NPR1*-like gene families across diverse citrus species provides compelling evidence of their pivotal role in citrus plants resistance mechanisms against biotic stress factors. This exploration not only enhances our understanding of the evolutionary relationships within these gene families but also offers promising avenues for addressing challenges in citrus production and elevating fruit quality. Our hypothesis proposes the targeted modification of the *NPR1* gene through CRISPR Cas9 technology, with the potential to yield a resilient *C. limon* variant resistant to Xac infection. Moreover, a thorough investigation into the roles played by SA and *NPR1* during biotic stress emerges as a promising direction for future research. By integrating modern natural breeding techniques—such as double haploids, hybridization, tissue culture, and backcrossing—we aim to fortify susceptibility-prone varieties, bolstering their resilience against both biotic and abiotic stresses. In this way, our holistic approach not only advances citrus resilience but also contributes to a broader understanding of plant resistance mechanisms, marking a significant step forward in the pursuit of sustainable and robust citrus cultivation.

## Data availability statement

The datasets presented in this study can be found in online repositories. The names of the repository/repositories and accession number(s) can be found in the article/supplementary material.

## Author contributions

MA: Conceptualization, Methodology, Writing – original draft. MS: Conceptualization, Data curation, Writing – review & editing. MZH: Conceptualization, Data curation, Visualization, Writing – review & editing. AS: Formal analysis, Investigation, Methodology, Writing – review & editing. PA: Project administration, Validation, Writing – review & editing. TA: Funding acquisition, Supervision, Validation, Writing – review & editing. ZK: Conceptualization, Formal analysis, Resources, Writing – review & editing. SS: Conceptualization, Data curation, Software, Writing – review & editing. MH: Investigation, Methodology, Writing – review & editing. MAS: Funding acquisition, Supervision, Visualization, Writing – review & editing. MJ: Funding acquisition, Visualization, Writing – review & editing. GA: Formal analysis, Funding acquisition, Investigation, Visualization, Writing – review & editing. MM: Data curation, Investigation, Methodology, Writing – review & editing. IS: Funding acquisition, Validation, Writing – review & editing.
